# Optimizing root architecture with nitrogen fertilization to improve nitrogen accumulation and yield in soybean

**DOI:** 10.3389/fpls.2026.1752272

**Published:** 2026-01-30

**Authors:** Yaxin Xu, Jianxin Zhang, Quantong Gao, Cong Wang

**Affiliations:** College of Agriculture, Xinjiang Agricultural University, Urumqi, China

**Keywords:** nitrogen fertilizer, nitrogen use efficiency, root structure, soybean, yield

## Abstract

**Introduction:**

Xinjiang is a high-yielding region for soybean in China, but issues such as low nitrogen use efficiency limit yield. Optimizing nitrogen fertilization strategies can effectively alleviate these problems.

**Methods:**

A two-year field experiment was conducted during the 2022 and 2023 growing seasons using a split-plot design. Two soybean cultivars, the low-yielding Xindadou 27 and the high-yielding Xinnongdou 2, were planted in the main plots. Four nitrogen application rates were applied in the subplots: 0, 120, 180, and 240 kg ha^-1^.

**Results:**

The application of 180 kg ha^-1^ nitrogen significantly increased root dry weight density, length density, and surface area density in the 0–60 cm soil layer, mainly through increases in the 0–20 cm soil layer. This treatment also enhanced the activities of key nitrogen metabolism enzymes (NR and GS/GOGAT) in roots, promoting nitrogen uptake and translocation to shoots, which increased both yield and shoot nitrogen accumulation. The higher accumulation rate and longer duration under the 180 kg N ha^-1^ application rate resulted in the highest root nitrogen accumulation. In contrast, a nitrogen application rate of 240 kg N ha^-1^ inhibited root growth, disrupted root nitrogen metabolism, and reduced root nitrogen accumulation. Structural equation modeling confirmed that root growth parameters have a positive influence on root nitrogen accumulation.

**Discussion:**

This study demonstrates that application of 180 kg N ha^-1^ at the beginning pod stage promotes root development, improves NUE and yield for spring soybean in Xinjiang. It is recommended as a sustainable high-yield practice for the region.

## 1 Introduction

As a major high-yielding soybean region in China, Xinjiang achieved a record soybean yield of 7126.2 kg ha^-1^ in 2025. The average soybean yield in China was only 1995 kg ha^-1^ (Li and Yang, 2025). This large gap highlights the substantial potential for increasing soybean yield in China. In the Xinjiang high-yield cultivation system, advanced mulched drip irrigation technology and precise nitrogen input have been instrumental in achieving this record yield (Zheng et al., 2021). However, excessive nitrogen application can limit soybean nitrogen use efficiency (He et al., 2025). Nitrogen is a key element regulating both root growth and yield formation in soybean (He et al., 2025). As the primary organ for nitrogen uptake, root system growth directly determines nitrogen acquisition efficiency and utilization potential. This ultimately affects yield potential (Walch-Liu et al., 2005). Thus, developing nitrogen management strategies that optimize root growth and enhance nitrogen absorption, translocation, and utilization efficiency is of great practical importance. Achieving both maximum nitrogen use efficiency and yield is crucial for sustainable agricultural practices and efficient soybean production.

The morphological structure of plant roots in the soil reflects their potential capacity for absorbing water and nitrogen, thereby supporting crop metabolism and growth (Dai et al., 2014). It has been demonstrated that an optimal root system architecture is conducive to the accumulation of nitrogen in the aboveground parts of the plant, thereby contributing to the formation of a high yield (Rotundo et al., 2014; Niu et al., 2020). Research indicates that high-yielding soybean varieties generally possess more fine roots, greater root length density, and deeper soil distribution—advantages that directly determine the crop’s nutrient acquisition potential (Jones and Jacobsen, 2005; Lynch and Brown, 2011; Symeou et al., 2012). Crop root architecture is closely related to the soil environment, particularly the supply of soil nitrogen. Appropriate nitrogen application promotes root extension into deeper soil layers and delays root senescence. This process not only facilitates the efficient allocation of photosynthetic products to reproductive organs but also promotes biomass and nitrogen accumulation in these organs (Jiang et al., 2017), ultimately enhancing nitrogen use efficiency (Fehr et al., 1971; Xu et al., 2012). Under nitrogen-deficient conditions, plant nitrogen metabolism significantly declines, markedly downregulating the glutamine synthetase (GS)/glutamate synthase (GOGAT) cycle, which accelerates leaf senescence and shortens the duration of crop photosynthesis (Takahashi et al., 2005), thereby inhibiting plant growth and limiting yield potential. Previous studies have shown that soybean yield under zero nitrogen treatment is 11% lower than that under nitrogen fertilizer application (Cafaro La Menza et al., 2017). In contrast, excessive nitrogen application leads to ammonium nitrogen accumulation in the soil, significantly reduces nitrate reductase (NR) and GS activities, causes root damage or even degeneration, and impairs soybean symbiotic nitrogen fixation (Guan et al., 2016). Extensive studies have confirmed significant positive correlations among soybean root architecture, nitrogen accumulation, and yield (Jin et al., 2006; He et al., 2017). Thus, optimizing soybean root architecture through rational nitrogen application is a crucial approach to enhancing nitrogen absorption efficiency and achieving high yields.

High-yielding soybean demands substantial nitrogen, requiring approximately 300 kg of nitrogen per 3 t ha^-^¹ of grain produced (Hungria, 2006; Salvagiotti et al., 2008; Tamagno et al., 2017). Notably, studies indicate that about 50% of the nitrogen in mature soybean grains derives from soil and fertilizer nitrogen (Salvagiotti et al., 2008). If nitrogen assimilation during the grain-filling stage is constrained by insufficient soil nitrogen supply or reduced symbiotic nitrogen fixation, plants are highly susceptible to nitrogen deficiency. This can lead to decreased pod number, increased abortion rate, and ultimately pose a serious threat to grain yield (Ortez et al., 2019). To achieve the synergistic maximization of nitrogen use efficiency and yield in soybean, it is essential to thoroughly investigate nitrogen supply patterns during reproductive growth and the interaction mechanisms between root growth, metabolism, and nitrogen absorption and utilization.

Under mulch drip irrigation, plastic mulch has significantly improved the temperature and moisture conditions in the soybean rhizosphere, promoting root growth and facilitating high-yield formation in soybeans (Wang et al., 2000). Currently, the influence patterns of nitrogen application rates on root architecture, nitrogen uptake in spring soybeans, and their relationship with yield formation in mulch drip irrigation areas of Xinjiang have not been reported. Under drip irrigation with plastic mulch, this study established different nitrogen application levels to elucidate the relationships among soybean root growth and metabolism, nitrogen uptake and utilization, and yield. We hypothesized that excessive nitrogen application at the beginning pod stage⁠ would inhibit root growth, suppress the GS/GOGAT pathway, and nitrogen translocation to shoots, thereby reducing soybean yield. Our results provide new insights into efficient nitrogen use and the regulation of precision nitrogen application for soybean in arid regions.

## 2 Materials and methods

### 2.1 Experimental field and meteorological conditions

**Figure 1:**
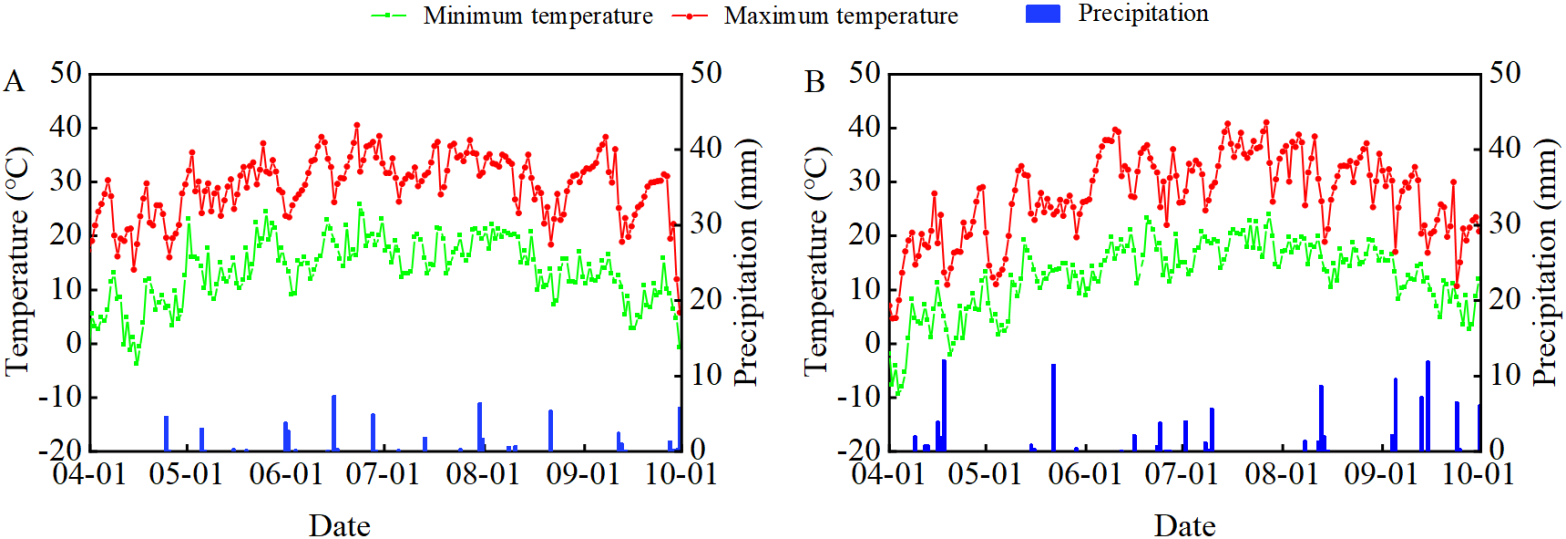
Daily minimum temperature, maximum temperature and rainfall from April to October in 2022 **(A)** and 2023 **(B)**.

The experiment was carried out in 2022–2023 at the Sanping Experimental Farm (116°41′E, 39°91′N; Urumqi, Xinjiang, China). Meteorological data (temperature and precipitation) for the soybean growing seasons are shown in **Figure 1**. The soil is classified as sandy loam, with initial properties as follows: organic matter content 13.8 g kg^-1^, total N 0.82 g kg^-1^, mineral N 56.65 mg kg^-1^, available P 14.1 mg kg^-1^, and available K 200.6 mg kg^-1^.

### 2.2 Experimental design and crop husbandry

The field experiment followed a split-plot arrangement over two consecutive growing seasons. The main plots consisted of two soybean cultivars: Xindadou 27 (a low-yielding cultivar) and Xinnongdou 2 (a high-yielding cultivar). The subplots comprised four nitrogen application levels: 0 (N_0_), 120 (N_120_), 180 (N_180_), and 240 kg ha^-1^ (N_240_). Among these, 0 kg N ha^-1^ served as the nitrogen-free control; 240 kg N ha^-1^ represented the conventional high-yield application rate used by local farmers under plastic mulching with drip irrigation; 180 kg N ha^-1^ was identified as the optimized threshold based on preliminary experiments, which maintained yield potential while significantly improving nitrogen use efficiency; and 120 kg N ha^-1^ represented a moderate nitrogen reduction treatment. All nitrogen fertilizer was applied in a single dose at the beginning pod stage via drip irrigation. Each plot measured 48 m² (4.8 m × 10 m). Each treatment was replicated three times, with a 2 m buffer zone between adjacent plots. Before tillage, superphosphate (containing 19% P_2_O_5_) was applied at 300 kg ha^-^¹. Soybeans were sown on April 27 and harvested from September 6 to 26. The experiment was conducted over two consecutive years. A uniform row spacing of 40 cm was used with a planting density of 33×10^4^ plants ha^-^¹. The field was covered with 140 cm wide black plastic mulch, and drip irrigation tapes were laid beneath the mulch at 40 cm intervals (Φ16 mm with a discharge rate of 2.5–3.5 L h^-1^). Irrigation was conducted annually from June 17–27 to August 5–15, once every 10–13 days, totaling six events with each irrigation applying 600–700 m³ ha^-1^. The total irrigation volume was 3900 m³ ha^-1^. Potassium dihydrogen phosphate (containing 34% K_2_O and 52% P_2_O_5_) was applied through fertigation. The total nutrient application rates were 51 kg K_2_O ha^-1^ and 78 kg P_2_O_5_ ha^-1^, applied in a 1:2 ratio at the initial flowering stage and the beginning of the pod stage, respectively. Two manual weeding were performed during the soybean growing season. All other management practices were consistent with conventional field production.

### 2.3 Root architecture

Root sampling was carried out at five key growth stages: full flowering stage⁠ (R_2_), full pod stage (R_4_), beginning pod stage (R_5_), full seed stage (R_6_), and full maturity stage (R_8_). For each treatment, the profile trench method was used for sampling. The experiment employed a planting pattern with four rows under a single plastic film. To systematically control the influence of root overlap between adjacent plants, all root samples were collected from the two middle rows. The specific sampling location was centered on a single plant selected from the middle row of plants. From this center point, sampling extended 10 cm along the row direction in both directions (total length 20 cm) and 20 cm perpendicular to the row direction in both directions (total width 40 cm). The excavation depth was 20 cm, resulting in a single-layer soil sampling volume of 16,000 cm^3^. This volume was determined based on the local sandy loam soil texture and the characteristic that soybean roots are primarily distributed in the 0–60 cm soil layer, ensuring both representativeness of the root samples and practicality for field operation and subsequent root separation. Root samples were collected from the 0–20 cm, 20–40 cm, and 40–60 cm soil layers separately. They were placed on a 2 mm mesh sieve and rinsed with gently flowing water. All root residues retained on the sieve were collected (pre-experiment verification indicated root loss during this operation was minimal, not affecting data accuracy). The roots were then sorted, re-screened, and washed, and the taproots and lateral roots were separated. The root samples were placed in a transparent glass box filled with water. Each root was carefully extended and flattened with forceps to avoid overlap and crossing. Before scanning, the WinRHIZO-2004a system was calibrated using its standard root images to ensure accurate measurement of root length and surface area. The root samples were then scanned using the root scanning analyzer to obtain the relevant parameters. After scanning, the root samples were placed in a 105°C oven for 30 minutes to deactivate enzyme activity, then dried at 80°C to a constant weight, and the root dry weight was recorded. Based on the sampling volume of each soil layer, the root dry weight density (RDD, Equation 1), root length density (RLD, Equation 2), and root surface area density (RSD, Equation 3) were calculated.

(1) $$RDD\;\left(g\;m^{-3}\right)\;=\frac{Root\;dry\;weight}{Soil\;volume}$$

(2) $$\;RLD\;\left(m\;m^{-3}\right)\;=\frac{Root\;length}{Soil\;volume}$$

(3) $$RSD\;\left(m^{2}m^{-3}\right)\;=\frac{Root\;surface\;area}{Soil\;volume}$$

### 2.4 Sample collection

During the full flowering stage (R_2_), full pod stage (R_4_), beginning pod stage (R_5_), full seed stage (R_6_), and full maturity stage (R_8_), ten representative plants were collected from each treatment. Root systems were carefully excavated and rinsed with tap water. Five plants were then separated into constituent organs and dried in an oven at 60°C until constant weight was achieved for biomass assessment. The dried plant material was ground to pass through a 1 mm sieve and stored under dry conditions. Roots from the remaining five plants were preserved at –80°C for later biochemical analysis.

### 2.5 Enzymatic activities

Fresh roots were extracted with an appropriate buffer, and the supernatant was collected for the determination of the following enzyme activities: nitrate reductase (NR), glutamine synthetase (GS), and glutamate synthase (GOGAT). NR activity was measured using the method of Ogawa et al (Ogawa et al. (1999)). with slight modifications: the reaction mixture contained 100 mM KNO_3_, 5 mM NaHCO_3_, 25 mM PBS, and 0.2 mM nicotinamide adenine dinucleotide (NADH). After the mixture had reacted thoroughly for 30 minutes, 250 μL of 1% sulfanilamide reagent and 250 μL of 1% α-naphthylamine reagent were sequentially added. A standard curve was prepared using sodium nitrite. GS activity was determined according to the method of O’Neal and Joy (O’Neal and Joy (1974)): the reaction system contained 50 mM Tris-HCl (pH 7.5), 4 mM ATP, 80 mM sodium glutamate, 30 mM MgSO_4_, 10 mM NH_2_OH, and 30 mM cysteine, with γ-glutamyl hydroxamate used to prepare the standard curve. GOGAT activity was assayed by the method of Singh and Srivastava (Singh and Srivastava (1986)): the reaction medium consisted of 100 mM potassium phosphate buffer (pH 7.6), 0.1% (*v*/*v*) 2-mercaptoethanol, 100 μM NADH, 2.5 mM 2-ketoglutarate, and 100 mM glutamine.

### 2.6 Total N accumulation amount and rate

Plant nitrogen content was determined using the Kjeldahl method: plant organs were digested with concentrated H_2_SO_4_ and H_2_O_2_, and nitrogen content was measured using a K9840 automatic Kjeldahl nitrogen analyzer. The total N accumulation in the samples was calculated using the following formula (Equation 4): 

(4) $$N\left(kg\;ha^{-1}\right)=DMA\;\left(kg\;ha^{-1}\right)\times Nc\left(\%\right)$$

Where DMA represents dry matter accumulation, and Nc represents the total nitrogen concentration.

The dynamics of total nitrogen accumulation in soybean were fitted using the Logistic equation as follows (Gao et al., 2021):

(5) $$Y=\frac{K}{1+ae^{bt}}$$

Where *t* is the days after emergence (DAE, d), *Y* is the plant biomass or total nitrogen amount (kg) at time *t*, K is the maximum biomass or total nitrogen accumulation (kg), and *a* and *b* are constants.

Using differential calculus on Equation 5 yields the following:

(6) $$t_{1}=\frac{1}{\text{b}}\text{ln}\left(\frac{2+\sqrt{3}}{\text{a}}\right),\;t_{2}=\frac{1}{\text{b}}\text{ln}\left(\frac{2-\sqrt{3}}{\text{a}}\right),\;\Delta t=t_{2}-t_{1},V_{t}=\frac{{\text{Y}}_{2}{\text{-Y}}_{1}}{{\text{t}}_{2}{\text{-t}}_{1}}$$

Where Δt is the fast accumulation period (FAP) of total nitrogen, t_1_ is the start time, t_2_ is the end time, and Vt is the mean accumulation rate during this FAP (Equation 6).

With reference to the methodology described by Mao et al. (2018) for quantifying biomass accumulation rates, the beta growth function (Equation 7) was employed to simulate the unimodal curve dynamics of total nitrogen accumulation in both roots and shoots.

(7) $$V=V_{m}(\frac{T_{e}-t}{T_{e}-T_{m}}){\left(\frac{t}{T_{m}}\right)}^{\frac{Tm}{T_{e}-T_{m}}}$$

Where V (kg ha^-1^ d^-1^) represents the total nitrogen accumulation rate at days after emergence (DAE) t (d); Te (d) denotes the termination time of accumulation, i.e., the accumulation duration; Tm (d) represents the occurrence time of the maximum accumulation rate Vm (kg ha^-1^ d^-1^). Tm and Te can be obtained by fitting the total nitrogen accumulation data using Equation 8, while Vm can be calculated using Equation 9.

(8) $$W=W_{\max}(1+\frac{T_{e}-t}{T_{e}-T_{m}}){\left(\frac{t}{T_{e}}\right)}^{\frac{T_{e}}{T_{e}-T_{m}}}$$

(9) $$V_{m}=W_{\max}(\frac{2T_{e}-T_{m}}{T_{e}(T_{e}-T_{m})}){\left(\frac{T_{m}}{T_{e}}\right)}^{\frac{T_{m}}{T_{e}-T_{m}}}$$

Where W (kg ha^-1^) and Wmax (kg ha^-1^) represent the total nitrogen accumulation at days after emergence t and the maximum total nitrogen accumulation, respectively.

### 2.7 Yield

At the full maturity stage, ten uniform, disease-free plants per treatment were sampled in three replicates to determine yield components, including pods per plant, grains per plant, and 100-grain weight. Grain yield was measured by harvesting a central area of 6.4 m² (1.6 m × 4 m) per plot, excluding border rows and the first meter at both ends, also with three replicates. The harvested grains were air-dried, weighed, and the yield was adjusted to a standard moisture content of 13%.

### 2.8 Nitrogen use efficiency

The nitrogen use efficiency (NUE) is calculated as follows (Equation 10):

(10) $$NUE=\frac{\text{Yield of nitrogen-treated plots }–\;\text{Yield of nitrogen-free plots}}{\text{Nitrogen application rate}}$$

### 2.9 Statistical analysis

Statistical analyses were performed using SPSS 20.0 (SPSS Inc., Chicago, IL, USA). One-way ANOVA was used to evaluate the effects of year, cultivar, growth stage, and nitrogen treatment on root architecture and enzyme activities. Two-way ANOVA was applied to examine the interactions between cultivar and nitrogen application rate on root architecture, nitrogen accumulation parameters, yield, and nitrogen use efficiency (NUE). Data are presented as means. Structural equation modeling (SEM) was implemented in IBM SPSS AMOS 26 to quantify the relationships among root architecture, nitrogen accumulation, and yield. Chi-square (χ^2^/df), root mean square error of approximation (RMSEA), standardized root mean square residual (SRMR), and comparative fit index (CFI) were used to evaluate the applicability of SEM (Noll et al., 2022). If an index did not meet the SEM evaluation criteria, the model was adjusted by adding significant relationships or removing non-significant relationships until all indices met the evaluation criteria.

## 3 Results

### 3.1 Root dry weight density

**Figure 2:**
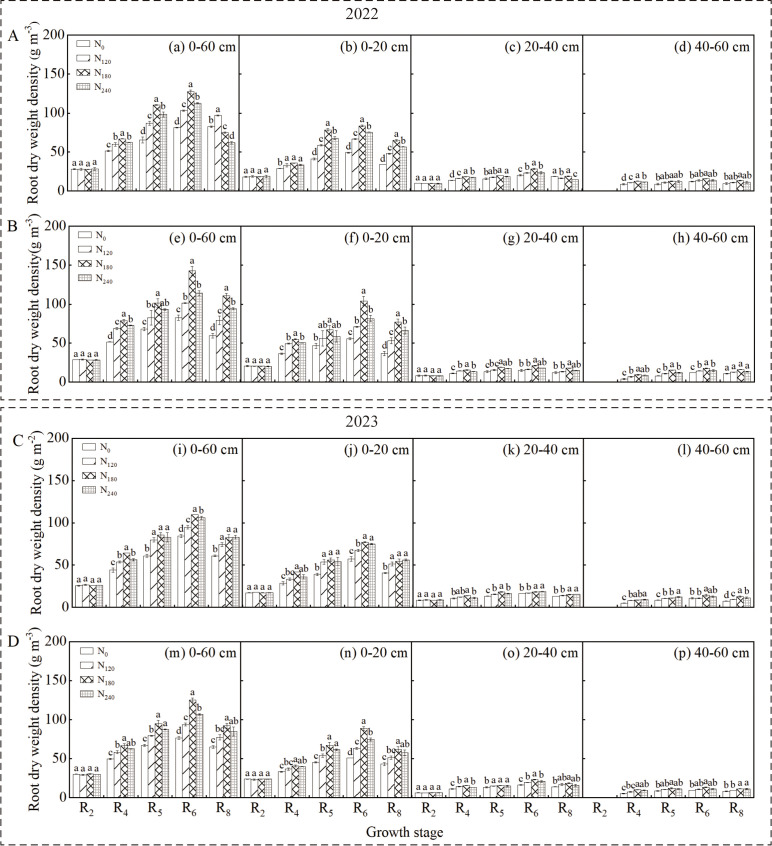
Effect of nitrogen application rate on soybean root dry weight density (RDD) in 2022 and 2023. Panels **(A, C)** represent the RDD of Xindadou 27, while Panels **(B, D)** represent that of Xinnongdou 2. Bars represent means and error bars standard error (*n* = 3). Different letters represent significant differences (*p* < 0.05) between treatments at the same growth stage.

Analysis of variance (**Table 1**) revealed that cultivar, nitrogen application rate, and their interaction significantly influenced root dry weight density (RDD) in the 0–60 cm soil layer during the R_4_~R_6_ stages in both growing seasons (*p* < 0.05). The RDD in the 0–60 cm and 0–20 cm layers exhibited a consistent pattern, initially increasing and then decreasing with growth progression, peaking at the R_6_ stage (**Figure 2**). Significant differences were observed among cultivars and nitrogen treatments (*p* < 0.05), with the N_180_ treatment consistently exhibiting the highest RDD. Compared to other treatments, the N_180_ treatment significantly increased RDD for both cultivars in both years. The trend in the 0–20 cm soil layer was consistent with that in the 0–60 cm layer.

**Table 1:** ANOVA of effects of cultivar and nitrogen application rate on root system architecture.

Soil depth	Source of variation	2022									
		R_4_			R_5_			R_6_		
		RDD	RLD	RSD	RDD	RLD	RSD	RDD	RLD	RSD
0-60	C	**	**	**	*	**	ns	**	**	*
	T	**	**	**	**	**	**	**	**	**
	C×T	**	**	ns	*	**	**	**	**	**
0-20	C	**	**	**	ns	**	*	**	**	**
	T	**	**	**	**	**	**	**	**	**
	C×T	**	*	*	**	*	**	**	**	**
20-40	C	**	**	**	**	**	*	**	ns	**
	T	**	**	**	**	**	**	**	**	**
	C×T	**	**	**	ns	**	ns	*	**	**
40-60	C	**	**	**	*	**	**	**	*	ns
	T	**	**	**	**	**	**	**	**	**
	C×T	**	*	**	**	**	**	ns	*	*
2023										
0-60	C	**	**	**	*	**	*	*	**	**
	T	**	**	**	**	**	**	**	**	**
	C×T	ns	**	**	*	**	**	**	**	**
0-20	C	**	**	ns	*	**	*	ns	**	**
	T	**	**	**	**	**	**	**	**	**
	C×T	**	**	**	**	**	**	**	**	**
20-40	C	**	**	**	ns	**	**	**	ns	**
	T	**	**	**	**	**	**	**	**	**
	C×T	*	**	**	**	ns	ns	**	**	**
40-60	C	ns	**	**	ns	*	**	**	*	*
	T	**	**	**	**	**	**	**	**	**
	C×T	**	ns	**	*	ns	ns	ns	**	**

Data are expressed as means (*n* = 3). ns, * and ** indicate nonsignificant, significant at 5% and 1% level, respectively.

### 3.2 Root length density

**Figure 3:**
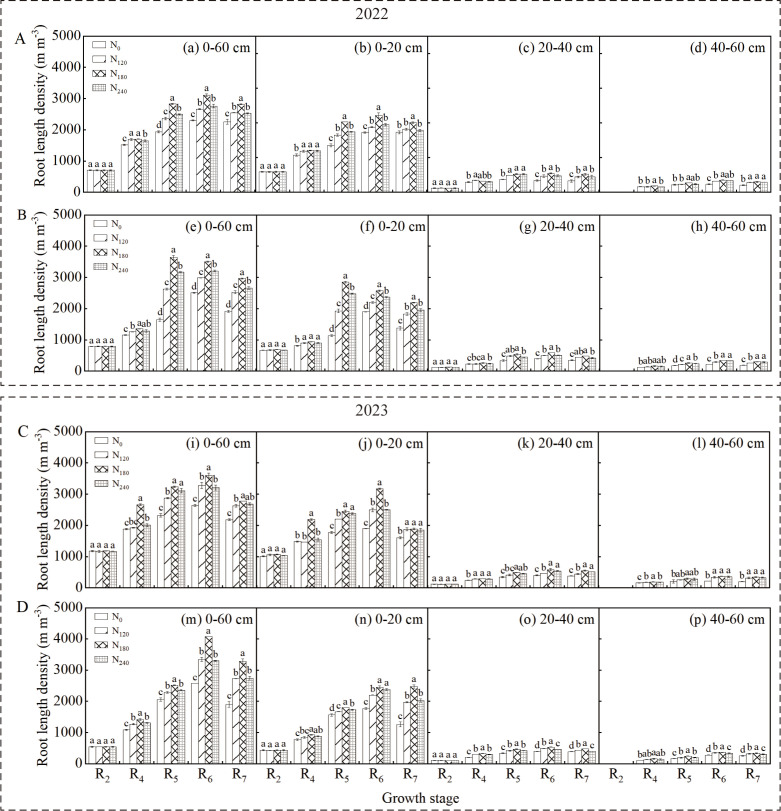
Effect of nitrogen application rate on soybean root length density (RLD) in 2022 and 2023. Panels **(A, C)** represent the RLD of Xindadou 27, while Panels **(B, D)** represent that of Xinnongdou 2. Bars represent means and error bars standard error (*n* = 3). Different letters represent significant differences (*p* < 0.05) between treatments at the same growth stage.

Analysis of variance (**Table 1**) indicated that cultivar, nitrogen application rate, and their interaction significantly affected soybean root length density (RLD) in the 0–60 cm soil layer during the R_4_~R_6_ stages in both growing seasons (*p* < 0.05). The RLD in both the 0–60 cm and 0–20 cm soil layers showed an initial increase followed by a decrease during the growth period, reaching maximum values at the R_6_ stage (**Figure 3**). Significant differences were observed among different cultivars and nitrogen treatments (*p* < 0.05), with the N_180_ treatment consistently exhibiting significantly higher RLD than other treatments across both cultivars and years. The changing pattern of RLD in the 0–20 cm soil layer was generally consistent with that in the 0–60 cm layer.

### 3.3 Root surface area density

**Figure 4:**
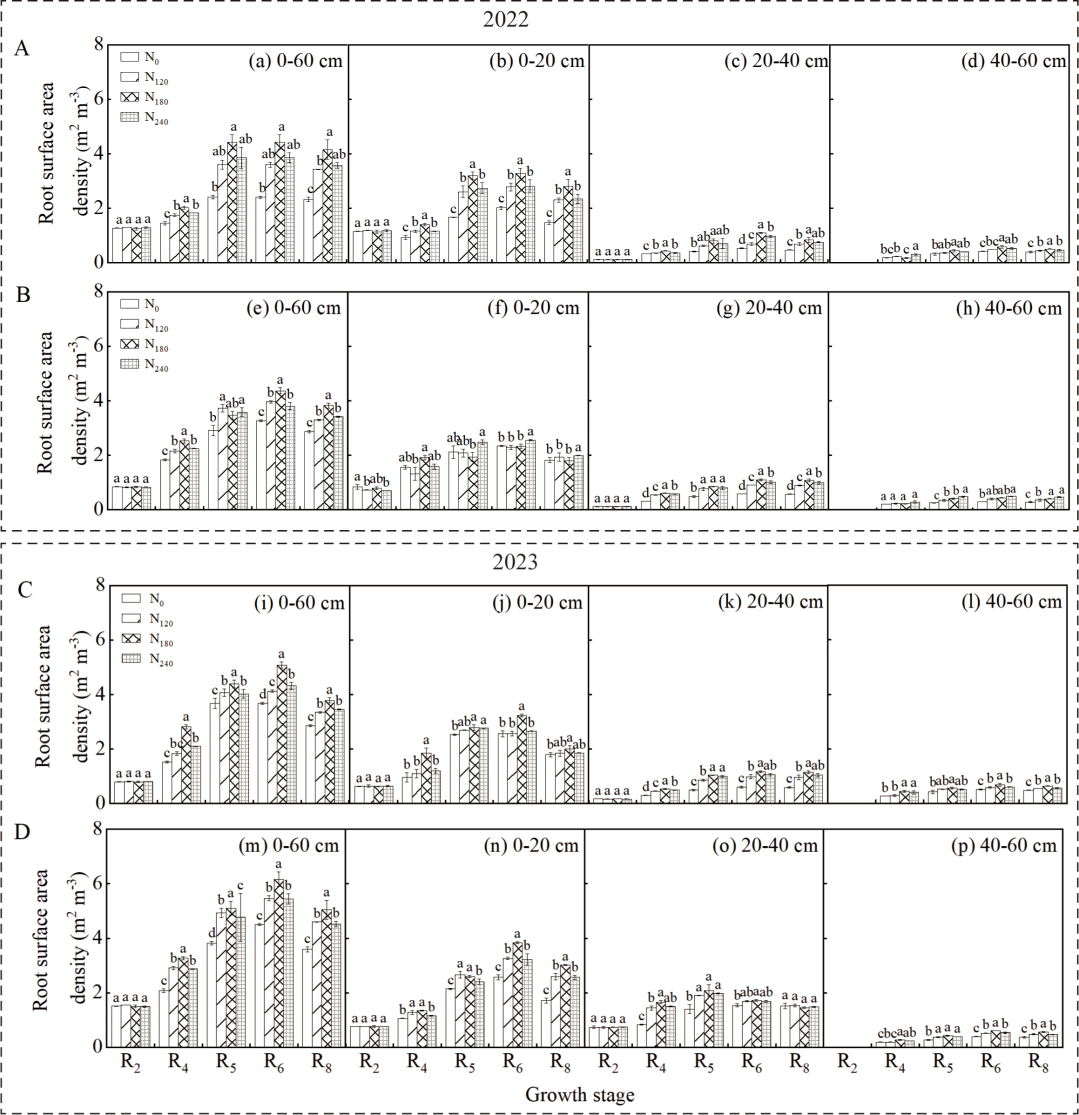
Effect of nitrogen application rate on soybean root surface area density (RSD) in 2022 and 2023. Panels **(A, C)** represent the RSD of Xindadou 27, while Panels **(B, D)** represent that of Xinnongdou 2. Bars represent means and error bars standard error (*n* = 3). Different letters represent significant differences (*p* < 0.05) between treatments at the same growth stage.

Analysis of variance revealed that cultivar, nitrogen application rate, and their interaction significantly affected soybean root surface area density (RSD) in the 0–60 cm soil layer during the R_4_~R_6_ stages across both growing seasons (*p* < 0.05). The RSD in both the 0–60 cm and 0–20 cm soil layers exhibited a pattern of initial increase followed by a decrease during the reproductive growth period, reaching maximum values at the R_6_ stage (**Figure 4**). Significant differences were observed among different cultivars and nitrogen treatments (*p* < 0.05), with the N180 treatment consistently showing significantly higher RSD than other treatments for both cultivars in both years. The RSD trend in the 0–20 cm soil layer was essentially consistent with that in the 0–60 cm layer.

### 3.4 Root enzyme activities

**Figure 5:**
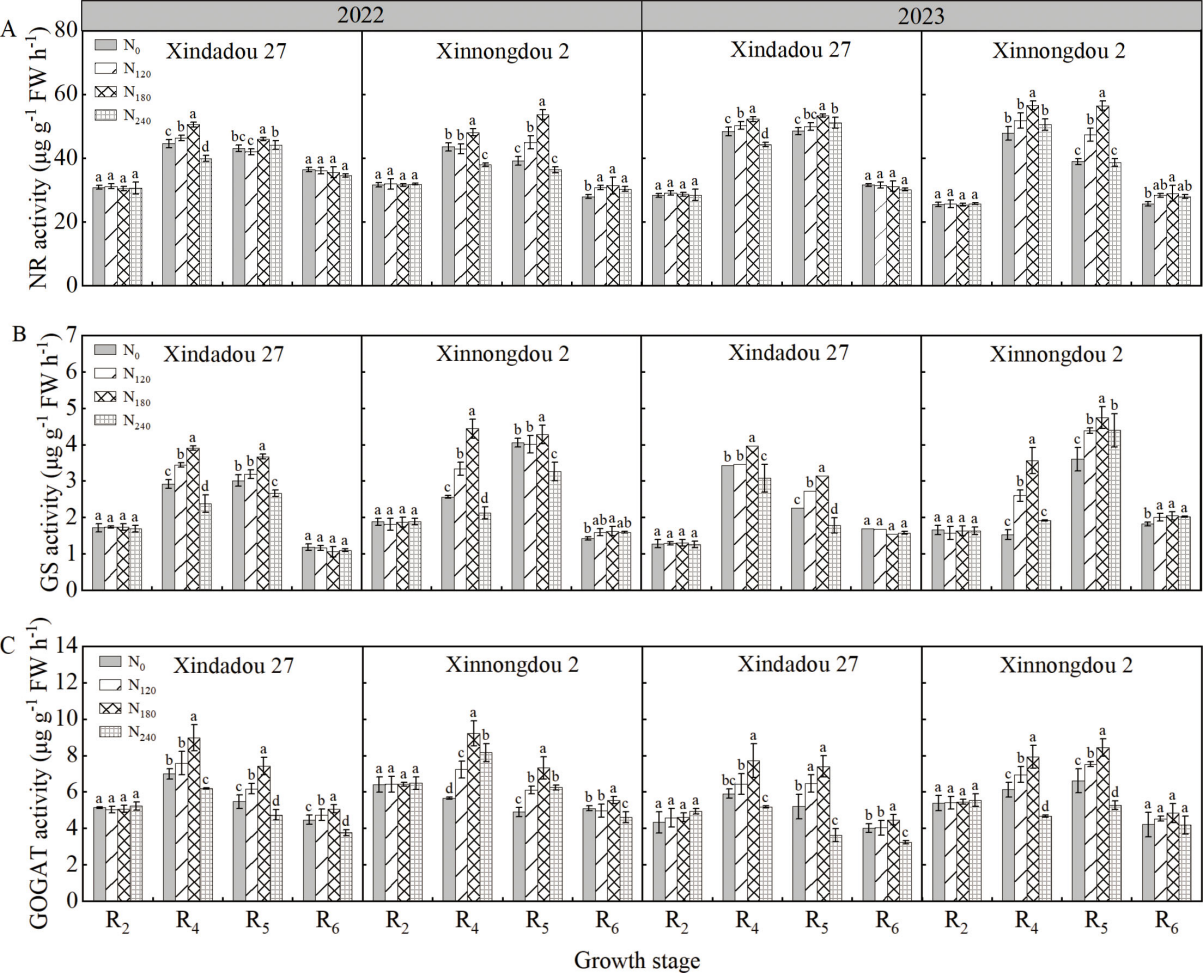
Effect of nitrogen application rate on soybean root enzyme activities in 2022 and 2023. Bars represent means and error bars standard error (*n* = 3). Different letters represent significant differences (*p* < 0.05) between treatments at the same growth stage.

As shown in **Figure 5**, during both the 2022 and 2023 growing seasons, the activities of NR, GS, and GOGAT in roots of Xindadou 27 and Xinnongdou 2 showed an initial increase followed by a decrease during the reproductive growth period, peaking at the R_4_ stage. Nitrogen application significantly increased the activities of root NR, GS, and GOGAT during the R_4_ and R_5_ stages (*p* < 0.05). The trends of enzyme activities in 2023 were generally consistent with those observed in 2022.

### 3.5 Root total nitrogen

**Figure 6:**
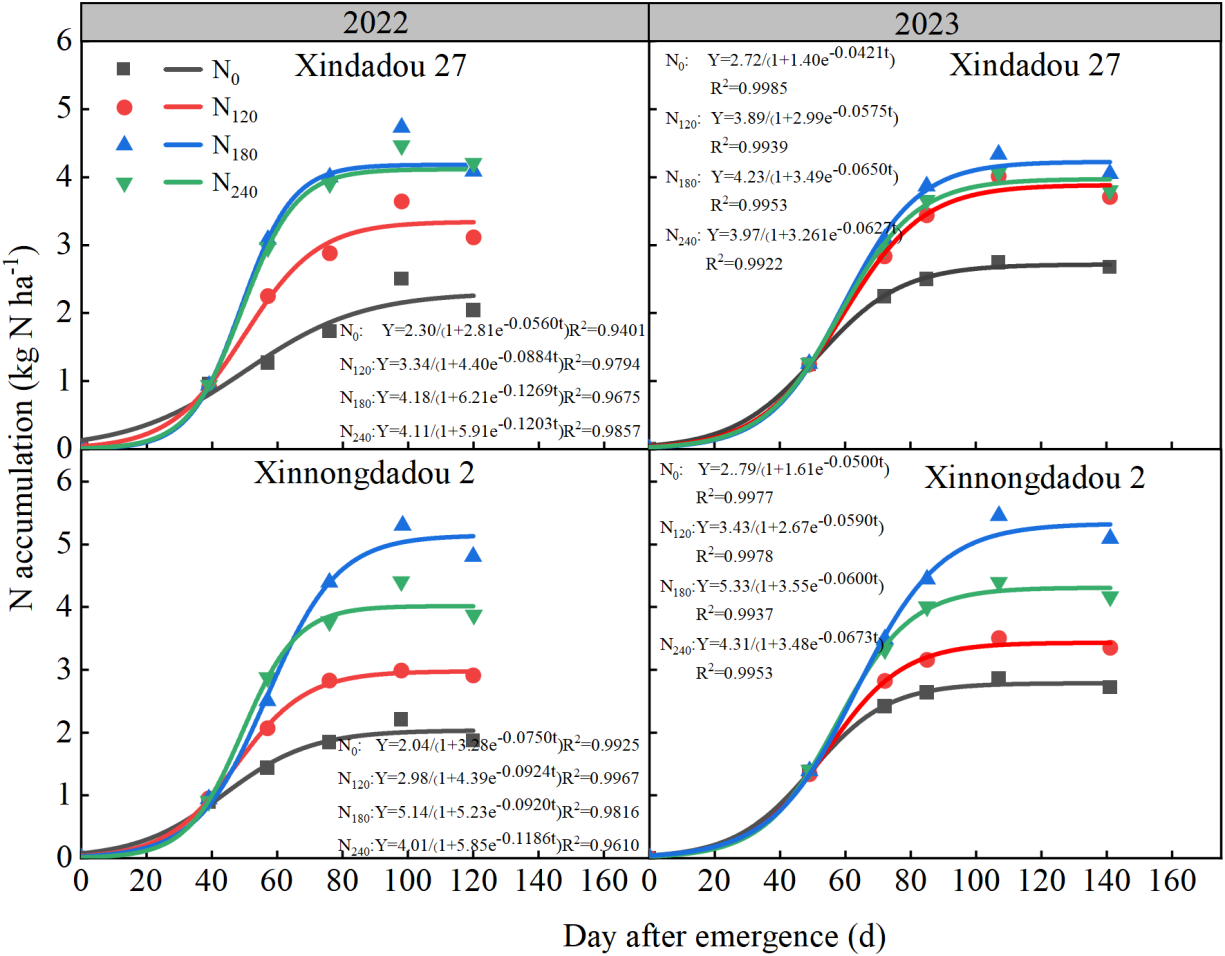
Effect of nitrogen application rate on soybean root nitrogen accumulation in 2022 and 2023. Points represent the measured mean values, while lines represent the logistic fitting.

**Figure 7:**
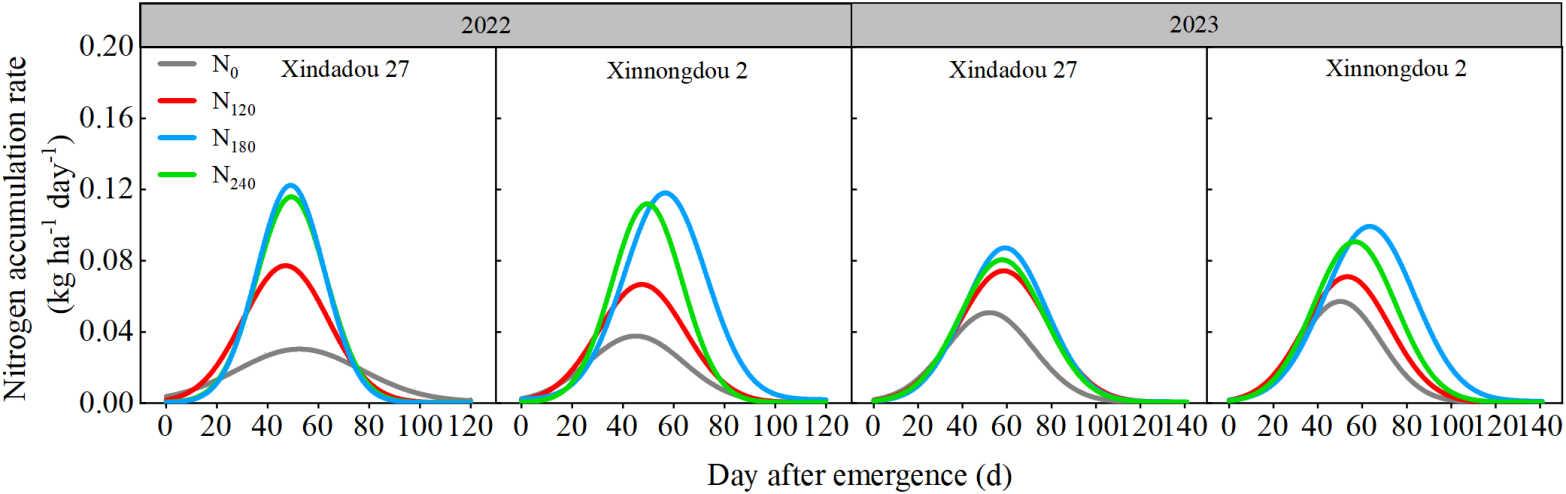
Effect of nitrogen application rate on soybean root nitrogen accumulation rate in 2022 and 2023 based on β-equation fitting.

During both the 2022 and 2023 growing seasons, the root nitrogen accumulation of both soybean cultivars exhibited a slowfast-slow pattern (**Figure 6**). The root nitrogen accumulation rate showed a typical single-peak curve, rising first and then falling (**Figure 7**). In 2022, as the nitrogen application rate increased, the total root nitrogen accumulation (K) and average accumulation rate (Vt) of both cultivars initially increased, and then decreased (**Table 2**). Nitrogen application significantly prolonged the fast accumulation period (FAP, Δt) of total root nitrogen. Compared to the N_0_ treatment, the K value and Vt values of Xindadou 27 in the N_120_, N_180_, and N_240_ treatments increased significantly by 42.55%~75.32% and 90.48%~214.29%, respectively; while those of Xinnongdou 2 increased by 45.37%~150.24% and 81.58%~213.16%, respectively.

**Table 2:** Effect of nitrogen application rate on parameters of total nitrogen accumulation in soybean roots.

Years	Cultivar (C)	Treatment (T)	K (kg ha^-1^)	t_1_ (d)	t_2_ (d)	Δt (d)	Vt (kg ha^-1^ d^-1^)
2022	Xindadou 27	N_0_	2.35g	26.57e	51.85d	25.27b	0.042f
		N_120_	3.35e	33.56c	60.99b	27.43ab	0.080c
		N_180_	4.18b	38.47b	67.30ab	28.82a	0.132a
		N_240_	4.12c	38.19b	60.11b	21.92d	0.124b
	Xinnongdou 2	N_0_	2.05h	25.50d	53.26c	27.76ab	0.038e
		N_120_	2.98f	33.22c	61.72b	28.51a	0.069d
		N_180_	5.13a	42.31a	70.79a	28.48a	0.119b
		N_240_	4.02d	38.18b	60.46b	22.29c	0.119b
		C	ns	*	ns	*	ns
	*P*	T	**	**	**	**	**
		C×T	**	**	**	**	**
2023	Xindadou 27	N_0_	2.72e	42.58b	65.95c	23.37d	0.051f
		N_120_	3.89c	46.07a	70.37bc	24.30d	0.077d
		N_180_	4.23b	41.84c	77.61b	35.77b	0.091b
		N_240_	3.97c	40.32d	69.86bc	29.54c	0.085c
	Xinnongdou 2	N_0_	2.79e	38.84d	69.75bc	30.91c	0.058e
		N_120_	3.43d	42.86ab	70.32bc	27.46c	0.073d
		N_180_	5.33a	42.28b	80.28a	38.00a	0.103a
		N_240_	4.31b	36.80d	75.28b	38.48a	0.096b
		C	**	*	*	ns	*
	*P*	T	**	**	**	**	**
		C×T	**	**	**	**	**

Data are expressed as means (*n* = 3). Different letters indicate a statistically significant level at *p*<0.05. ns, * and ** indicate nonsignificant, significant at 5% and 1% level, respectively.

### 3.6 Shoot nitrogen accumulation

**Figure 8:**
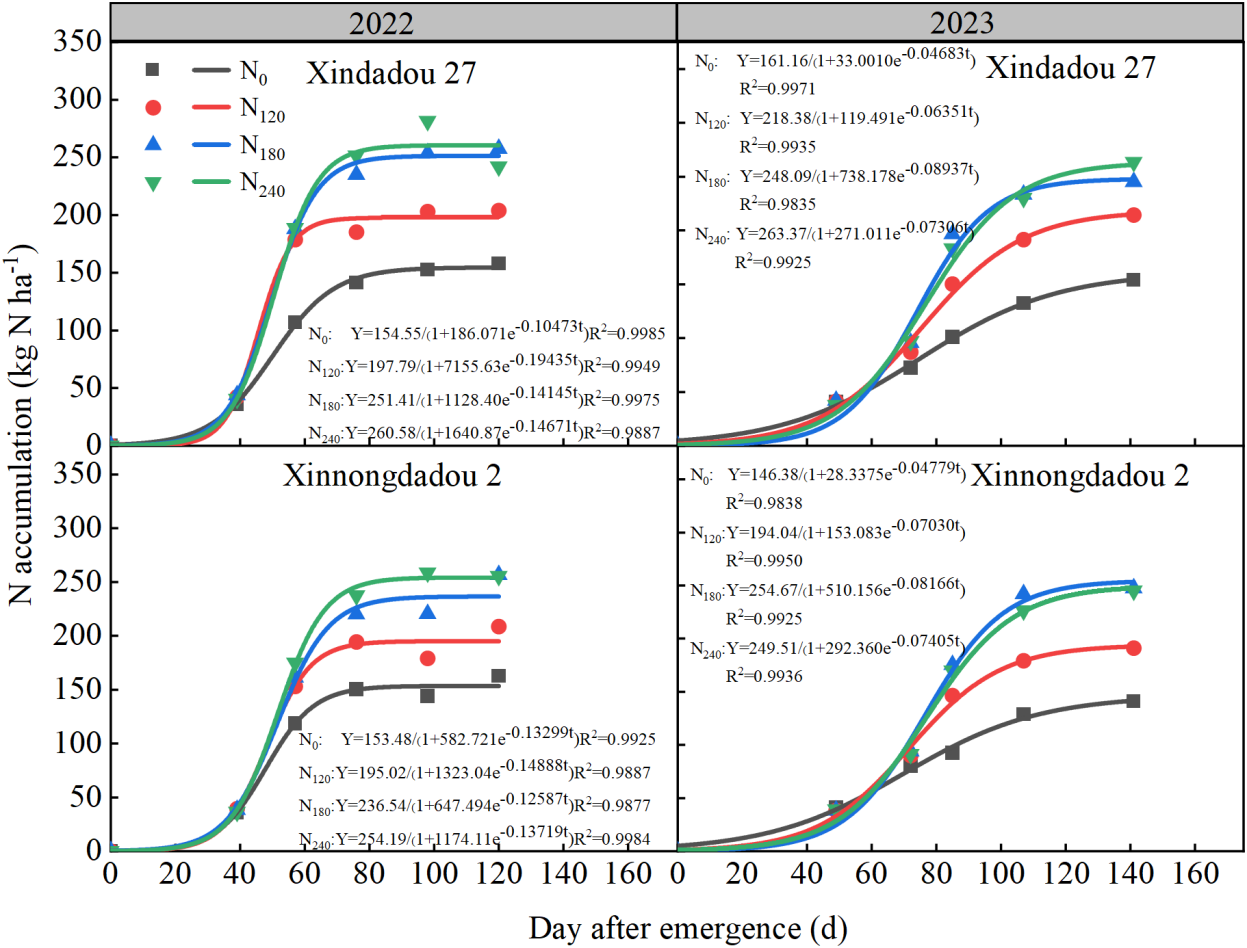
Effect of nitrogen application rate on soybean aboveground nitrogen accumulation in 2022 and 2023. Points represent the average of measurements, while the lines represent logistic fits.

**Figure 9:**
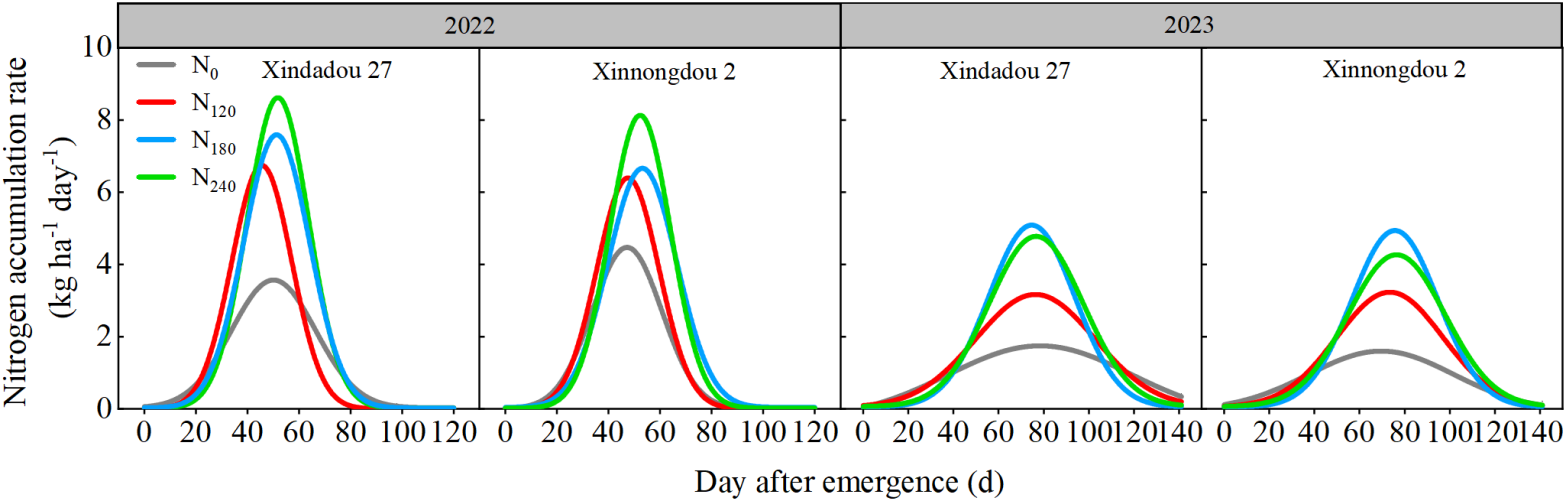
Effect of nitrogen application rate on soybean aboveground nitrogen accumulation rate in 2022 and 2023 based on β-equation fitting.

Over the 2022 and 2023 growing seasons, aboveground nitrogen accumulation in both soybean cultivars followed a typical sigmoidal curve (**Figure 8**). The aboveground nitrogen accumulation rate showed a typical single-peak curve, rising first and then falling (**Figure 9**). As shown in **Table 3**, key accumulation parameters increased with the application of nitrogen. Notably, the N_180_ treatment significantly enhanced the final accumulation potential (K), prolonging the rapid accumulation duration (Δt) by 23.97%~28.58% and substantially increasing the average accumulation rate (Vt) compared to the N0 control. This trend was consistent across both years, with the N_180_ rate consistently promoting the most favorable nitrogen uptake dynamics for each cultivar.

**Table 3:** Effect of nitrogen application rate on parameters of total nitrogen accumulation in soybean shoots.

Year	Cultivar	Treatment	K (kg ha^-1^)	t_1_ (d)	t_2_ (d)	Δt (d)	Vt (kg ha^-1^ d^-1^)
2022	Xindadou 27	N_0_	154.75e	37.22e	51.67e	14.45d	4.02d
		N_120_	197.84d	39.50d	54.14d	14.63d	8.93a
		N_180_	251.43b	40.42c	59.00b	18.58b	8.97a
		N_240_	260.62a	41.48ab	59.44ab	17.96c	9.58a
	Xinnongdou 2	N_0_	153.68e	37.97e	54.95d	16.98c	5.12c
		N_120_	194.99d	39.50d	57.11c	17.61c	7.40b
		N_180_	236.45c	41.07bc	62.12a	21.05a	7.55b
		N_240_	254.21ab	41.93a	61.13a	19.20ab	8.73a
		C	*	*	*	**	*
	*P*	T	**	**	**	**	**
		C×T	ns	ns	**	**	**
2023	Xindadou 27	N_0_	163.29e	45.63d	78.73d	33.10c	1.82e
		N_120_	219.04c	54.55b	96.42ab	41.88a	3.46d
		N_180_	248.42b	59.16a	98.78a	39.62a	5.54a
		N_240_	263.51a	58.74a	94.73ab	35.99b	4.85bc
	Xinnongdou 2	N_0_	148.23f	41.08e	73.48e	32.40d	1.67e
		N_120_	197.82d	52.91c	90.45c	37.55ab	3.43d
		N_180_	254.72ab	60.16a	93.48b	33.32d	5.19ab
		N_240_	249.56b	58.85a	94.5b	35.65b	4.61c
		**C**	*	**	*	*	*
	*P*	T	**	**	**	**	**
		C×T	*	**	**	*	ns

Data are expressed as means (*n* = 3). Different letters indicate a statistically significant level at *p*<0.05. ns, * and ** indicate nonsignificant, significant at 5% and 1% level, respectively.

### 3.7 Yield and nitrogen use efficiency

The ANOVA results (**Table 4**) indicated that both cultivar and nitrogen application rate significantly affected pods per plant, seeds per plant, 100-seed weight, yield, and nitrogen use efficiency (NUE). Yield consistently reached its peak under the N_180_ treatment for both cultivars across both years. Compared to N_0_, the yield increases for Xindadou 27 were 19.32% in 2022 and 25.57% in 2023, and for Xinnongdou 2, they were 24.92% in 2022 and 21.81% in 2023. This yield advantage at N_180_ was associated with a significant increase in both the number of pods per plant and the number of seeds per plant. Notably, while 100-seed weight often reached its maximum under the N_240_ treatment, this increase in individual seed mass did not lead to higher overall yield beyond the optimum achieved at N_180_. For NUE, both cultivars reached their maximum under N_180_ in 2022. In 2023, the highest values for Xindadou 27 and Xinnongdou 2 were 4.13 kg kg^-1^ under N_180_ and 4.88 kg kg^-1^ under N_120_, respectively.

**Table 4:** Effect of nitrogen application rate on yield and NUE. Data are expressed as means (*n* = 3).

Year	Cultivar	Treatment	Pods per plant	Seeds per plant	100-grain weight	Yield	NUE
(Y)	(C)	(T)			(g)	(kg ha^-1^)	
2022	Xindadou 27	N_0_	25.25e	77.85e	18.40c	3433.36f	
		N_120_	30.40d	85.9cd	19.67b	3721.68e	2.40c
		N_180_	37.60b	95.65b	19.87b	4096.68b	3.69b
		N_240_	33.95c	89.45c	20.94a	3857.54cd	1.77d
	Xinnongdou 2	N_0_	33.40c	79.40e	15.37e	3418.34f	
		N_120_	37.80b	89.40c	16.60d	3827.51d	3.41b
		N_180_	42.80a	99.95a	17.17d	4292.54a	4.86a
		N_240_	38.25b	93.35b	18.53c	3932.52c	2.14cd
	*P*	C	**	**	**	*	*
		T	**	**	**	**	**
		C×T	ns	ns	ns	*	*
2023	Xindadou 27	N_0_	28.40e	77.85e	18.44c	3525.94d	
		N_120_	32.95d	85.9cd	19.26b	3881.50c	2.96b
		N_180_	36.90c	95.65b	19.87ab	4021.50b	4.13a
		N_240_	34.00d	89.45c	20.29a	3911.13c	1.60b
	Xinnongdou 2	N_0_	37.60c	79.40e	16.58e	3810.39c	
		N_120_	41.80b	89.40c	17.54d	4396.32b	4.88a
		N_180_	47.65a	99.95a	18.00cd	4641.50a	4.62a
		N_240_	42.95b	93.35b	17.94cd	4333.35b	2.18b
	*P*	C	**	ns	**	*	*
		T	**	**	**	**	**
		C×T	ns	ns	ns	ns	*

Different letters indicate a statistically significant level at *p*<0.05. ns, * and ** indicate nonsignificant, significant at 5% and 1% level, respectively.

### 3.8 Structural equation model

**Figure 10:**
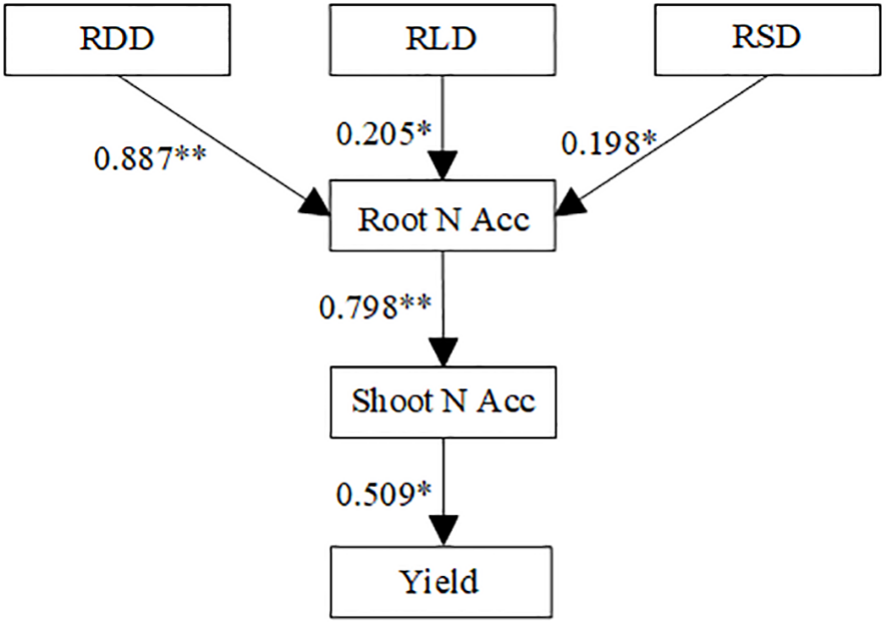
Structural equation modeling (SEM) of the relationships between root system architecture, nitrogen accumulation and yield at R6 stage in 2022 and 2023. Solid and dashed arrows indicate significant and nonsignificant path coefficients, respectively. RDD, root dry weight density; RLD, root length density; RSD, root surface area density; Root N Acc, nitrogen accumulation in root; Shoot N Acc, nitrogen in the shoot. Values above arrows represent standardized path coefficients. ** denote significance at the 0.01 probability levels, * denote significance at the 0.05 probability levels.

As shown in **Table 5**, all fit indices were within the acceptable range, with χ²/df=0.649, RMSEA = 0.029, SRMR = 0.031, and CFI = 1.000. All fit parameters of the initial model indicated a good fit. Structural equation modeling (SEM) was applied to evaluate the effects of root architecture and nitrogen accumulation on yield (**Figure 10**). Root dry weight density (RDD), root length density (RLD), and root surface area density (RSD) significantly and positively influenced root nitrogen accumulation, with path coefficients of 0.887, 0.205, and 0.198, respectively. Root nitrogen accumulation demonstrated a highly significant positive effect on aboveground nitrogen accumulation. Furthermore, aboveground nitrogen accumulation exhibited a significant positive effect on yield, with a path coefficient of 0.509.

**Table 5:** Fitting coefficients of yield and its driving factors in paddy soils using structural equation modeling analysis. χ², Chi-square test; df, degree of freedom; RMSEA, root mean square error of approximation; SRMR, standardized root mean square residual; CFI, comparative fit index.

Item	χ²/df	RMSEA	SRMR	CFI
Evaluation criterion	<3	<0.05	<0.05	close to 1
Result	0.649	0.029	0.031	1.000

## 4 Discussions

In high-yield environments, the external nitrogen supply serves as the primary approach to meet soybean nitrogen demand, with root morphology and distribution determining nitrogen acquisition the capacity (Luo et al., 2020; Suo et al., 2024). Extensive studies have shown that the R_3_~R_6_ stages, particularly the R_4_~R_5_ stages, represent the peak nitrogen demand stage in soybeans, whereas external nitrogen application during early flowering stages has no significant effect on seed yield (Moreno et al., 2018; Ortez et al., 2019; De Borja Reis et al., 2021). Therefore, in this study, nitrogen was applied at the R_3_ stage. As a result, during the R_2_ stage across two growing seasons, no significant differences were observed in root length density (RLD), root dry weight density (RDD), or root surface area density (RSD) in the 0–60 cm soil layer among treatments (**Figures 2**-**4**). However, as growth progressed to the R_4_~R_6_ stages, significant differences in these root parameters emerged (**Table 1**), with the N_180_ treatment exhibiting higher values than other treatments. Some studies suggest that plants enhance root biomass under low nitrogen conditions by increasing nutrient allocation to roots (Gao et al., 2015; Kramer-Walter and Laughlin, 2017). In contrast, this study found that root biomass did not increase under low nitrogen but rather increased with nitrogen application up to a certain level before declining significantly. Low nitrogen supply likely inhibited root development by failing to meet basic growth demands, particularly in the zero-nitrogen treatment. Excessive nitrogen fertilizer also negatively affected root growth. The inhibition of root growth by high nitrogen (240 kg ha^-^¹) was directly associated with the simultaneous decline in key nitrogen metabolic enzyme activities. The significant reductions in NR, GS, and GOGAT activity led to decreased nitrogen assimilation efficiency in the roots. This resulted in the accumulation of absorbed ammonium nitrogen, which potentially triggered ammonium toxicity (Zhang et al., 2017; Chen et al., 2020). Some research indicates that nitrogen deficiency can promote root elongation to enhance spatial nitrogen availability (Sun et al., 2020), whereas other studies suggest that optimal external nitrogen levels stimulate lateral root growth, with both extremely low and high nitrogen levels suppressing root development (Walch-Liu et al., 2006; Zhu et al., 2022). This study supports these conclusions: nitrogen application at 180 kg ha^-1^ significantly improved shallow root traits (RLD, RDD, RSD). At the R_6_ stage, the N_180_ treatment increased RLD in the 0–60 cm soil layer by 9.9%~36.5% for Xindadou 27 and 9.2%~58.0% for Xinnongdou 2, and root surface area by 14.5%~84.1% and 10.2%~36.9%, respectively, compared to other treatments. Furthermore, averaged over two years during the R_4_~R_6_ stages, Xinnongdou 2 showed higher RLD, RDD, and RSD than Xindadou 27, indicating that its more developed root system facilitates more efficient soil resource absorption and utilization (**Figure 10**).

Previous studies have indicated that crop root growth is closely related to nitrogen accumulation and seed yield (Cafaro La Menza et al., 2019). Low nitrogen stress reduces nitrogen content and yield in field-grown plants (Lawlor et al., 2001), but high-yielding cultivars demonstrate higher nitrogen accumulation and yield under low nitrogen conditions due to greater nitrogen uptake efficiency and more extensive root systems (Ju et al., 2015; Su et al., 2019). The present study observed that root GS and GOGAT activity peaked during the R_4_ stage, representing an active physiological adaptation by plants to meet the nitrogen demands of rapid pod and grain development (Zhou et al., 2023). The rapid formation of pods and grains during the R_4_ stage generates intense nitrogen demand, driving the root system to efficiently synthesize transportable nitrogen compounds such as glutamine through upregulation of the core nitrogen assimilation pathway GS/GOGAT. This phenomenon occurs under conditions of sufficient carbon skeleton supply, thereby ensuring nitrogen availability for grain development (Andrews et al., 2004; Lyu et al., 2024). This study observed that appropriate external nitrogen supply significantly promotes root nitrogen assimilation, thereby increasing nitrogen accumulation, while excessively high or low nitrogen levels inhibit this process. These effects correspond to the activity trends of key nitrogen assimilation enzymes (NR, GS, GOGAT), in agreement with the results reported by Xu et al. (2012). In this study, nitrogen accumulation in both roots and aboveground canopies followed a “slow-fast-slow” pattern across all treatments (**Figure 6**, **Tables 2**, **3**). Analysis of nitrogen accumulation parameters revealed significant effects of cultivar and nitrogen fertilizer (*p* < 0.05). Although the rapid accumulation period for root and canopy nitrogen in the N_0_ treatment started 3.5~16.8 d and 3.4~19.1 d earlier, respectively, compared to other treatments, it also ended 10.5~17.5 d and 2.2~21.0 d earlier, resulting in shorter rapid accumulation durations by 0.8~12.4 d and 4.1~8.8 d, respectively. Meanwhile, the maximum accumulation rates for root and canopy nitrogen in the N0 treatment were 42.9%~68.2% and 41.4%~67.8% lower than other treatments. Consequently, root nitrogen accumulation followed N_180_>N_240_>N_120_>N_0_, while canopy nitrogen accumulation showed N_240_>N_180_>N_120_>N_0_. While N_240_ treatment resulted in the greatest canopy nitrogen accumulation, this could potentially lead to excessive vegetative growth and subsequent yield reduction. In comparison with Xinnongdou 2, Xindadou 27 demonstrated higher canopy nitrogen accumulation under conditions of elevated nitrogen availability. However, this was accompanied by a substantial reduction in pods per plant, resulting in a consequent decline in yield.

Nitrogen application rate serves as a primary method for regulating plant nitrogen accumulation and distribution, playing a crucial role in plant growth and yield potential (Anas et al., 2020; Wang et al., 2024). Both excessively high and low nitrogen supply levels can reduce soybean yield. In this study, the maximum soybean yield was achieved at the nitrogen application rate of 180 kg ha^-1^, primarily due to increased pod and seed numbers, as an appropriate nitrogen supply during reproductive growth can compensate for plant nitrogen deficiency (Tamagno, 2018). Yield reduction under high nitrogen application was mainly attributed to enhanced vegetative growth, which inhibited flower and pod formation (Li et al., 2024). Comparative analysis between cultivars revealed that Xinnongdou 2 yielded significantly higher than Xindadou 27. This difference was primarily attributed to the more developed root system of Xinnongdou 2, which contributed to its stronger soil nutrient uptake capacity. Subsequently, through more efficient nitrogen conversion and allocation, a greater proportion of the absorbed nitrogen was directed toward pod and seed formation, enabling the plants to achieve higher yield and NUE. Therefore, in future breeding for high yield and high efficiency, emphasis could be placed on selecting traits related to superior root architecture and high pod and seed number potential. This provides important phenotypic and physiological foundations for developing new soybean varieties that combine high yield with efficient nutrient use. We conducted a structural equation modelling analysis to evaluate the relationships among root morphological traits, nitrogen accumulation and yield at the R_5_ stage. The analysis revealed that RDD, RLD, and RSD positively regulated root nitrogen accumulation with path coefficients of 0.887, 0.205, and 0.198, respectively, explaining why superior root morphological characteristics facilitate nitrogen absorption and utilization from soil (Li et al., 2016). Root nitrogen accumulation positively regulated aboveground canopy nitrogen accumulation, which in turn positively influenced yield, accounting for yield variations among different soybean cultivars (Kiba and Krapp, 2016).

## 5 Conclusion

This study demonstrates that nitrogen application at 180 kg·ha^-^¹ during the beginning pod stage significantly promoted root development in both soybean cultivars, substantially increasing root length density, root dry weight density, and root surface area density in the 0–60 cm soil layer, while enhancing the activity of key nitrogen assimilation enzymes (NR, GS/GOGAT) in the roots. In contrast, either excessive or insufficient nitrogen supply inhibited root growth and reduced root mass. Root morphological traits exhibited a significant positive regulatory effect on plant nitrogen accumulation, promoting efficient nitrogen uptake and translocation, thereby increasing soybean yield. The high-yielding cultivar Xinnongdou 2, with its greater root mass and nitrogen uptake capacity, not only ensured the material supply required for high yield but also effectively mitigated the risks of growth imbalance associated with excessive nitrogen application.

## Data Availability

The raw data supporting the conclusions of this article will be made available by the authors, without undue reservation.

## References

[B1] Anas M. Liao F. Verma K. K. Sarwar M. A. Mahmood A. Chen Z.-L. . (2020). Fate of nitrogen in agriculture and environment: agronomic, eco-physiological and molecular approaches to improve nitrogen use efficiency. Biol. Res. 53, 47. doi: 10.1186/s40659-020-00312-4, PMID: 33066819 PMC7565752

[B2] Andrews M. Lea P. J. Raven J. A. Lindsey K. (2004). Can genetic manipulation of plant nitrogen assimilation enzymes result in increased crop yield and greater N‐use efficiency? An assessment. Ann. Appl. Biol. 145, 25–40. doi: 10.1111/j.1744-7348.2004.tb00356.x

[B3] Cafaro La Menza N. Monzon J. P. Specht J. E. Grassini P. (2017). Is soybean yield limited by nitrogen supply? Field Crops Res. 213, 204–212. doi: 10.1016/j.fcr.2017.08.009

[B4] Cafaro La Menza N. Monzon J. P. Specht J. E. Lindquist J. L. Arkebauer T. J. Graef G. . (2019). Nitrogen limitation in high-yield soybean: Seed yield, N accumulation, and N-use efficiency. Field Crops Res. 237, 74–81. doi: 10.1016/j.fcr.2019.04.009

[B5] Chen J. Liu L. Wang Z. Zhang Y. Sun H. Song S. . (2020). Nitrogen fertilization increases root growth and coordinates the root–shoot relationship in cotton. Front. Plant Sci. 11. doi: 10.3389/fpls.2020.00880, PMID: 32655605 PMC7324761

[B6] Dai X. Xiao L. Jia D. Kong H. Wang Y. Li C. . (2014). Increased plant density of winter wheat can enhance nitrogen–uptake from deep soil. Plant Soil 384, 141–152. doi: 10.1007/s11104-014-2190-x

[B7] De Borja Reis A. F. Moro Rosso L. Purcell L. C. Naeve S. Casteel S. N. Kovács P. . (2021). Environmental factors associated with nitrogen fixation prediction in soybean. Front. Plant Sci. 12. doi: 10.3389/fpls.2021.675410, PMID: 34211487 PMC8239404

[B8] Fehr W. R. Caviness C. E. Burmood D. T. Pennington J. S. (1971). Stage of development descriptions for soybeans, glycine max (L.) merrill. Crop Sci. 11, 929–931. doi: 10.2135/cropsci1971.0011183X001100060051x

[B9] Gao K. Chen F. J. Yuan L. X. (2015). A comprehensive analysis of root morphological changes and nitrogen allocation in maize in response to low nitrogen stress. Plant Cell Environ. 38, 740–750. doi: 10.1111/pce.12439, PMID: 25159094

[B10] Gao H. Li N. Li J. Khan A. Ahmad I. Wang Y. . (2021). Improving boll capsule wall, subtending leaves anatomy and photosynthetic capacity can increase seed cotton yield under limited drip irrigation systems. Ind. Crops Products 161, 113214. doi: 10.1016/j.indcrop.2020.113214

[B11] Guan M. de Bang T. C. Pedersen C. (2016). Cytosolic glutamine synthetase Gln1; 2 is the main isozyme contributing to GS1 activity and can be up-regulated to relieve ammonium toxicity. Plant Physiol. 171, 1921–1933. doi: 10.1104/pp.16.01195, PMID: 27231101 PMC4936538

[B12] He H. Guo R. Deng K. Pang S. Liu J. (2025). Water and nitrogen conservation enhance summer soybean (Glycine max) yield via improved photosynthesis and pod formation traits. Front. Sustain. Food Syst. 9. doi: 10.3389/fsufs.2025.1614074

[B13] He J. Jin Y. Du Y.-L. Wang T. Turner N. C. Yang R.-P. . (2017). Genotypic variation in yield, yield components, root morphology and architecture, in soybean in relation to water and phosphorus supply. Front. Plant Sci. 8. doi: 10.3389/fpls.2017.01499, PMID: 28912792 PMC5583600

[B14] Hungria M. (2006). Contribution of biological nitrogen fixation to the N nutrition of grain crops in the tropics: the success of soybean (Glycine max L. Merr.) in South America. Nitrogen Nutr. Plant productivity 2, 43–93. doi: 10.1007/1-4020-3544-6_3

[B15] Jiang S. Sun J. Tian Z. Hu H. Michel E. J. S. Gao J. . (2017). Root extension and nitrate transporter up-regulation induced by nitrogen deficiency improves nitrogen status and plant growth at the seedling stage of winter wheat (Triticum aestivum L.). Environ. Exp. Bot. 141, 28–40. doi: 10.1016/j.envexpbot.2017.06.006

[B16] Jin J. Wang G. Liu X. Pan X. Herbert S. J. Tang C. (2006). Interaction between phosphorus nutrition and drought on grain yield, and assimilation of phosphorus and nitrogen in two soybean cultivars differing in protein concentration in grains. J. Plant Nutr. 29, 1433–1449. doi: 10.1080/01904160600837089

[B17] Jones C. Jacobsen J. (2005). Response of malt barley to phosphorus fertilization under drought conditions. J. Plant Nutr. 28, 1605–1617. doi: 10.1080/01904160500203531

[B18] Ju C. Buresh R. J. Wang Z. Zhang H. Liu L. Yang J. . (2015). Root and shoot traits for rice varieties with higher grain yield and higher nitrogen use efficiency at lower nitrogen rates application. Field Crops Res. 175, 47–55. doi: 10.1016/j.fcr.2015.02.007

[B19] Kiba T. Krapp A. (2016). Plant nitrogen acquisition under low availability: regulation of uptake and root architecture. Plant Cell Physiol. 57, 707–714. doi: 10.1093/pcp/pcw052, PMID: 27025887 PMC4836452

[B20] Kramer-Walter K. R. Laughlin D. C. (2017). Root nutrient concentration and biomass allocation are more plastic than morphological traits in response to nutrient limitation. Plant Soil 416, 539–550. doi: 10.1007/s11104-017-3234-9

[B21] Lawlor D. W. Lemaire G. Gastal F. (2001). “ Nitrogen, plant growth and crop yield,” in Plant nitrogen. Eds. Lea P. J. Morot-Gaudry J.-F. ( Springer Berlin Heidelberg, Berlin, Heidelberg), 343–367. doi: 10.1007/978-3-662-04064-5_13

[B22] Li X. Yang R. (2025). Xinjiang Production and Construction Corps Sets New National High-Yield Record for Spring-Sown Soybeans with Average Yield Reaching 475.08 kg per Mu. People.cn. Available at: http://xj.people.com.cn/n2/2025/1008/c186332-41373833.html.

[B23] Li X. Zeng R. Liao H. (2016). Improving crop nutrient efficiency through root architecture modifications. JIPB 58, 193–202. doi: 10.1111/jipb.12434, PMID: 26460087

[B24] Li M. Zhang K. Liu J. Nizam Ul Din G. (2024). Nitrogen addition mitigates drought by promoting soybean (Glycine Max (Linn.) Merr) flowering and podding and affecting related enzyme activities. Agriculture 14, 852. doi: 10.3390/agriculture14060852

[B25] Luo L. Zhang Y. Xu G. (2020). How does nitrogen shape plant architecture? J. Exp. Bot. 71, 4415–4427. doi: 10.1093/jxb/eraa187, PMID: 32279073 PMC7475096

[B26] Lynch J. P. Brown K. M. (2011). Topsoil foraging – an architectural adaptation of plants to low phosphorus availability. Plant Soil 237, 225–237. doi: 10.1023/A:1013324727040

[B27] Lyu X. Wang X. Li S. Yan C. Ma C. Zhao S. . (2024). Responses of metabolic pathways in soybean nodules and roots to long-term indirect nitrogen supply by dual-root system. Plant Soil 501, 241–265. doi: 10.1007/s11104-024-06518-9

[B28] Mao L. Zhang L. Sun X. van der Werf W. Evers J. B. Zhao X. . (2018). Use of the beta growth function to quantitatively characterize the effects of plant density and a growth regulator on growth and biomass partitioning in cotton. Field Crops Res. 224, 28–36. doi: 10.1016/j.fcr.2018.04.017

[B29] Moreno G. Albrecht A. J. P. Albrecht L. P. Junior C. P. Pivetta L. A. Tessele A. . (2018). Application of nitrogen fertilizer in high-demand stages of soybean and its effects on yield performance. Aust. J. Crop Sci. 12, 16–21. doi: 10.21475/ajcs.18.12.01.pne507

[B30] Niu L. Yan Y. Hou P. Bai W. Zhao R. Wang Y. . (2020). Influence of plastic film mulching and planting density on yield, leaf anatomy, and root characteristics of maize on the Loess Plateau. Crop J. 8, 548–564. doi: 10.1016/j.cj.2019.12.002

[B31] O’Neal D. Joy K. W. (1974). Glutamine synthetase of pea leaves. Plant Physiol. 54, 773–779. doi: 10.1104/pp.54.5.773, PMID: 16658970 PMC366601

[B32] Ogawa T. Fukuoka H. Yano H. Ohkawa Y. (1999). Relationships between nitrite reductase activity and genotype-dependent callus growth in rice cell cultures. Plant Cell Rep. 18, 576–581. doi: 10.1007/s002990050625

[B33] Ortez O. A. Tamagno S. Salvagiotti F. Prasad P. V. V. Ciampitti I. A. (2019). Soybean nitrogen sources and demand during the seed-filling period. Agron. J. 111, 1779–1787. doi: 10.2134/agronj2018.10.0656

[B34] Rotundo J. L. Borrás L. De Bruin J. Pedersen P. (2014). Soybean nitrogen uptake and utilization in Argentina and United States cultivars. Crop Sci. 54, 1153–1165. doi: 10.2135/cropsci2013.09.0618

[B35] Salvagiotti F. Cassman K. G. Specht J. E. Walters D. T. Weiss A. Dobermann A. (2008). Nitrogen uptake, fixation and response to fertilizer N in soybeans: A review. Field Crops Res. 108, 1–13. doi: 10.1016/j.fcr.2008.03.001

[B36] Singh R. P. Srivastava H. S. (1986). Increase in glutamate synthase (NADH) activity in maize seedlings in response to nitrate and ammonium nitrogen. Physiologia Plantarum 66, 413–416. doi: 10.1111/j.1399-3054.1986.tb05944.x

[B37] Su W. Kamran M. Xie J. Meng X. Han Q. Liu T. . (2019). Shoot and root traits of summer maize hybrid varieties with higher grain yields and higher nitrogen use efficiency at low nitrogen application rates. PeerJ 7, e7294. doi: 10.7717/peerj.7294, PMID: 31341742 PMC6637931

[B38] Sun X. Chen F. Yuan L. Mi G. (2020). The physiological mechanism underlying root elongation in response to nitrogen deficiency in crop plants. Planta 251, 84. doi: 10.1007/s00425-020-03376-4, PMID: 32189077

[B39] Suo R. Wang M. Zhao T. (2024). Contribution of photosynthetic, root and phenotypic traits to soybean plant height. Sustainability 16, 2886. doi: 10.3390/su16072886

[B40] Symeou V. Edwards S. A. Kyriazakis I. (2012). Modeling digestibility of dietary phosphorus in growing and finish pigs1. J. Anim. Sci. 90, 59–61. doi: 10.2527/jas.53804, PMID: 23365282

[B41] Takahashi M. Nakayama N. Arihara J. (2005). Plant nitrogen levels and photosynthesis in the supernodulating soybean (Glycine max L. Merr.) cultivar ‘Sakukei 4.’. Plant Production Sci. 8, 412–418. doi: 10.1626/pps.8.412

[B42] Tamagno S. (2018). Interplay between nitrogen fertilizer and biological nitrogen fixation in soybean: implications on seed yield and biomass allocation. Sci. Rep. 8, 17502. doi: 10.1038/s41598-018-35672-1, PMID: 30504907 PMC6269449

[B43] Tamagno S. Balboa G. R. Assefa Y. Kovács P. Casteel S. N. Salvagiotti F. . (2017). Nutrient partitioning and stoichiometry in soybean: A synthesis-analysis. Field Crops Res. 200, 18–27. doi: 10.1016/j.fcr.2016.09.019

[B44] Walch-Liu P. Filleur S. Gan Y. Forde B. G. (2005). Signaling mechanisms integrating root and shoot responses to changes in the nitrogen supply. Photosynth Res. 83, 239–250. doi: 10.1007/s11120-004-2080-9, PMID: 16143854

[B45] Walch-Liu P. Ivanov I. I. Filleur S. Gan Y. Remans T. Forde B. G. (2006). Nitrogen regulation of root branching. Ann. Bot. 97, 875–881. doi: 10.1093/aob/mcj601, PMID: 16339770 PMC2803407

[B46] Wang D. Shannon M. C. Grieve C. M. Yates S. R. (2000). Soil water and temperature regimes in drip and sprinkler irrigation, and implications to soybean emergence. Agricultural Water Management 43, 15–28. doi: 10.1016/S0378-3774(99)00057-8

[B47] Wang Q. Li S. Li J. Huang D. (2024). The utilization and roles of nitrogen in plants. Forests 15, 1191. doi: 10.3390/f15071191

[B48] Xu G. Fan X. Miller A. J. (2012). Plant Nitrogen Assimilation and Use Efficiency. Annu. Rev. Plant Biol. 63, 153–182. doi: 10.1146/annurev-arplant-042811-105532, PMID: 22224450

[B49] Zhang Z. Xiong S. Wei Y. Meng X. Wang X. Ma X. (2017). The role of glutamine synthetase isozymes in enhancing nitrogen use efficiency of N-efficient winter wheat. Sci. Rep. 7, 1000. doi: 10.1038/s41598-017-01071-1, PMID: 28428629 PMC5430530

[B50] Zheng C. Wang R. Zhou X. Li C. Dou X. (2021). Effects of mulch and irrigation regimes on water distribution and root competition in an apple–soybean intercropping system in Loess Plateau, China. Agric. Water Manage. 246, 106656. doi: 10.1016/j.agwat.2020.106656

[B51] Zhou H. Liu Y. Mu B. Wang F. Feng N. Zheng D. (2023). Nitrogen limitation affects carbon and nitrogen metabolism in mung bean (Vigna radiata L.). J. Plant Physiol. 290, 154105. doi: 10.1016/j.jplph.2023.154105, PMID: 37871476

[B52] Zhu L. Liu L. Sun H. Zhang K. Zhang Y. Li A. . (2022). Low nitrogen supply inhibits root growth but prolongs lateral root lifespan in cotton. Ind. Crops Products 189, 115733. doi: 10.1016/j.indcrop.2022.115733

